# Efficacy of continuous preperitoneal ropivacaine infusion in women with cesarean section: A prospective, randomized controlled, single blinded study

**DOI:** 10.1016/j.heliyon.2024.e39608

**Published:** 2024-10-21

**Authors:** Woo Jeng Kim, Eui-Jin Cho, Gyul Jung, In Seon Hwang, Jong Bun Kim, Yoonho Kim, Hee Joung Lee, Yeon-Hee Kim

**Affiliations:** a Department of Obstetrics and Gynecology, Incheon St. Mary's Hospital, The Catholic University of Korea, South Korea; b Department of Obstetrics and Gynecology, Uijeongbu St. Mary's Hospital, The Catholic University of Korea, South Korea; c Department of Anesthesiology, Uijeongbu St. Mary's Hospital, The Catholic University of Korea, South Korea

**Keywords:** Cesarean section, Continuous ropivacaine infusion, Local anesthetic, Postoperative

## Abstract

**Background:**

Adequate postoperative pain management after cesarean section is important for the prognosis of both mother and infant. However, excessive prescription of opioid analgesics has become a concern. This study evaluated the efficacy of local continuous subfascial infusion of ropivacaine in relieving postoperative pain and reducing opioid requirements in postpartum women with cesarean section.

**Methods:**

Seventy eligible women undergoing cesarean section were randomly allocated to the ropivacaine and the normal saline group for continuous subfascial wound infiltration. All patients received additional fentanyl through an intravenous patient-controlled analgesia pump. Pain score using the visual analog scale, opioid consumption through pump, and requirements for other analgesics were postoperatively measured within 8 h, 1 day, and 2 days after surgery. Statistical analysis was performed with independent t-tests for continuous variables and Chi-square tests for categorical variables. Paired Wilcoxon and student's t-tests were used for paired samples.

**Results:**

Sixty-nine patients (35 in the study and 34 in the control group) were analyzed. The mean VAS scores were lower in the study group all three periods, with significance achieved at day 2 (2.74 ± 0.95 versus 3.41 ± 1.33, *p* = 0.028). The intravenous fentanyl consumptions were significantly lower in the study group at all three periods. Total administration of additional non-opioid analgesics including ketorolac, propacetamol, and pethidine was higher in the control group.

**Conclusions:**

Continuous subfascial ropivacaine infusion is effective in relieving pain and reducing opioid-based analgesia and other analgesics requirements.

## Highlights

• Proper pain management of women after giving birth is vital for the prognosis of them and their infants.
• Continuous preperitoneal ropivacaine infusion effectively reduced postoperative pain scores of women with cesarean section.
• Patients with Continuous preperitoneal ropivacaine infusion dispensed lower amounts of intravenous opioids.

## 1 Introduction

The cesarean section rate is rapidly rising globally due to the increased rate of multifetal pregnancy, advanced maternal age, and maternal request. It is estimated that over 25 million cesarean sections are conducted globally each year [1]. It is reported that more patients choose cesarean delivery to avoid the pain during vaginal birth; however, the pain after giving birth is more severe and prolonged in patients with cesarean sections compared to patients with vaginal delivery.

Optimal pain management after cesarean section is important for both mother and infant prognosis. Inadequate treatment of pain after cesarean section can delay the return to normal daily activities, impair mother–child bonding, impact maternal psychological wellbeing, and reduce breastfeeding [2]. Furthermore, inadequate treatment can also contribute to hyperalgesia, persistent pain, and greater opioid usage [3].

In general, systemic opioid analgesics are effective in managing postoperative pain, they often lead to adverse effects including nausea, vomiting, itching, and dizziness. Moreover, common serious complications include respiratory suppression, difficulty in urination, paralytic ileus and increased intracranial pressure, and. The safety concern of infant exposure to opioids during breastfeeding exists in obstetric patients. In recent years, the pathway for elective cesarean section and the practical intervention for perioperative care were formulated based on reviews and meta-analyses. Pain management was a critical part of the prognosis of the mother and infant, and multimodal analgesia is currently advocated to avoid and reduce systemic opioid usage [4].

Continuous wound infiltration by local anesthetics (LA) delivers postoperative analgesia as a multimodal approach. Manufactured by I Flow Corp. (Lake Forest, CA, USA), the ON-Q pain management system (referred to as the ON-Q pump) delivers continuous local analgesia directly to the surgical wound site within the abdomen. The effect of continuous wound infiltration by LA on pain relief in various surgical sites has been demonstrated in previous studies [[4], [5], [6], [7]].

Since opioid-based intravenous patient-controlled analgesia (IV-PCA) and the On-Q system are commonly used together in the clinic, it is difficult to accurately evaluate the pain relief effect of the On-Q system and its role in reducing the opioid dose. Opioid analgesics are often prescribed for pain management immediately after cesarean section [2,3,8]. As opioids are weakly basic, lipophilic, and have a low molecular weight, they can be easily transferred from the mother's blood to breast milk [9]. However, the extent to which these drugs are released into breastmilk is unknown. Therefore, it is important to reduce opioid usage for pain management and adopt an effective multimodal approach.

The aim of this randomized placebo-controlled single-blinded study was to assess the effectiveness of 0.5 % ropivacaine compared to 0.9 % saline delivered by the ON-Q pump in controlling pain after surgery. Both groups also used the fentanyl-based IV-PCA system for additional pain management. The pain measurements were assessed using the visual analogue scale (VAS). The secondary endpoints were the dispensed amount of IV-PCA, use of additional rescue analgesics and occurrence of postoperative nausea and vomiting (PONV) and dizziness.

## 2 Methods

### 2.1 Patients

The study was a prospective randomized single-blinded controlled trial conducted in the outpatient clinic at the department of Obstetrics and Gynecology in Uijeongbu St. Mary's Hospital, the Catholic University of Korea between 2021 and 2022. The hospital ethical review board approved the study (approval number UC20DISI0042).

Pregnant patients undergoing scheduled cesarean section under either general anesthesia or spinal anesthesia by single operator (YH Kim) were screened for recruitment in this study. The inclusion criteria required participants to be between the ages of 19 and 49, and American Society of Anesthesiologists (ASA) physical status of II. The exclusion criteria included individuals with ASA physical status of III or higher, history of alcohol or drug abuse, those on daily opioid or glucocorticoid therapy, those allergic to ropivacaine or opioids, those with psychiatric disorders, non-Korean speakers. Written informed consent was obtained from the patients, and the study was carried out in accordance with the principles of the Declaration of Helsinki.

Patients were randomly assigned to either the active treatment group or the placebo group. For allocation sequence, a nurse, who was not involved in the trial, generated a sequence of 70 numbers using a computer soft program. Based on this sequence, she prepared 70 opaque envelopes, with 35 containing local anesthetic and 35 containing saline. Patient recruitment was conducted by the lead researcher (YH Kim) during outpatient visits. After explaining the study and the randomization process to eligible patients, based on the inclusion and exclusion criteria, those who agreed to participate were enrolled in the study.

Once a patient consented to participate in the study, they were assigned a number in the order of their consent. When the patient arrived in the operating room for their giving birth, the corresponding envelope was opened, and the assigned treatment was administered via the OnQ pump. All patients were blinded to their group allocation throughout the study.

Patients subsequently underwent wound infiltration with 270 mL of 0.5 % ropivacaine (Naropin®, AstraZeneca, Copenhagen, Denmark) (study group) or 270 mL of 0.9 % saline (control group).

### 2.2 ON-Q pain management system for wound infiltration

The ON-Q pain management system comprises an elastomeric pump containing a solution of local analgesia (total volume, 270 mL), consisting of 200 mL of ropivacaine (150 mg/20 mL) mixed with 70 mL of 0.9 % normal saline. The pump delivers analgesics through a catheter inserted into the surgical incision site within the abdomen, delivering a 0.5 % ropivacaine solution at a rate of 2–5 mL/h through a multi-holed catheter, maintaining a consistent pressure of 10 psi. The catheter was placed by inserting 20-gauge guiding needles into the preperitoneal subfascial layer of the surgical incision after suturing up to the peritoneum. We removed the catheters after 48 h of placement.

### 2.3 Post-operative analgesia with intravenous patient-controlled analgesia

In all the patients, IV-PCA (Accufuser Plus, WooYoung Medicals, Korea) was administered containing 1000 ㎍ of fentanyl citrate and 16 mg ondansetron hydrochloride in 0.9 % normal saline in total of 120 mL. IV-PCA was initiated immediately after surgery. IV-PCA infusion is maintained continuously at a basal flow rate of 2 mL/h and patients can administer a bolus of 0.5 mL by pressing the button once when experiencing pain. Following each bolus injection, there is a 15-min lock-out period. The following doses of 1 g per 6 h of paracetamol (Profa infusion, Dai Han Pharm. Co., Seoul, Korea), 30 mg per 8 h of ketorolac (Ketocin inj., Myungmoon Pharm. Co., Seoul, Korea), and 25 mg per 12 h of pethidine hydrochloride (Meperidine, JEIL Pharmaceuticals, Yongin-si, Gyeonggi-do, Korea) were systematically administered intravenously during the first 48 h starting from arrival in the post-anesthesia care unit. After 48 h, the PCA device and all other intravenous therapies were stopped.

### 2.4 Assessment of outcomes

Demographic characteristics and obstetric findings including gestational age, indication of cesarean section, and obstetric complications and pregnancy outcomes including neonate birth weight and Apgar score were collected. The patients were followed up at three time points (within 8 h and at postoperative day 1 and day 2) to investigate the study outcomes. The intensity of pain was assessed using the VAS scale which involves a 10-cm horizontal line with endpoints marked as “no pain” and “the worst possible pain.”

The fentanyl-based IV-PCA doses that were dispensed were checked at every visit. When the VAS index of pain was >4, additional analgesics were administered intravenously on demand in the following sequential order based on whether the patient asked again for analgesics, or the pain was not successfully controlled: 1 g propacetamol; 30 mg ketorolac; and 25 mg pethidine hydrochloride.

The number of additional analgesics dispensed since the last follow-up visit was measured each time. Adverse symptoms related to analgesics including postoperative nausea and vomiting (PONV) and dizziness.

### 2.5 Statistical analysis

After initial descriptive analysis, statistical analysis for continuous variables was performed using either the Wilcoxon Rank-Sum test or the independent sample *t*-test, depending on the normality assumption of the data. The Wilcoxon Rank-Sum test was used when the data did not meet the assumption of normality, while the independent sample *t*-test was employed for normally distributed data. Categorical variables were analyzed using either Fisher's exact test or Chi-square test, depending on the sample size and the presence of expected cell counts <5. Fisher's exact test was used for small sample sizes or when the expected cell count was <5, whereas the Chi-square test was applied for larger sample sizes. For paired samples, the Wilcoxon signed-rank test or paired student's t-test was used, depending on the distributional characteristics of the data. The Wilcoxon signed-rank test was used for non-normally distributed data, whereas the paired student's t-test was applied for normally distributed data. A two-tailed *P* value < 0.05 was considered statistically significant. All statistical analyses were performed using R version 4.2.2 (R Foundation for Statistical Computing).

The sample size calculation was based on the total dose of fentanyl infused through the PCA device for 48 h postoperatively. A power analysis suggested that a minimum group size of 30 patients would give a 90 % power to detect a 25 % reduction in fentanyl-based PCA dispensed (1000㎍ fentanyl citrate for saline control; 750 ㎍ fentanyl citrate for study group) at POD 2, using a one-tailed, two-sample *t*-test and assuming a significance level of 0.05, a standard deviation of 300, and normally distributed data. To factor in the expected drop-out rate, a sample size for each group was estimated to be about 30–35 patients.

## 3 Results

**Fig. 1:**
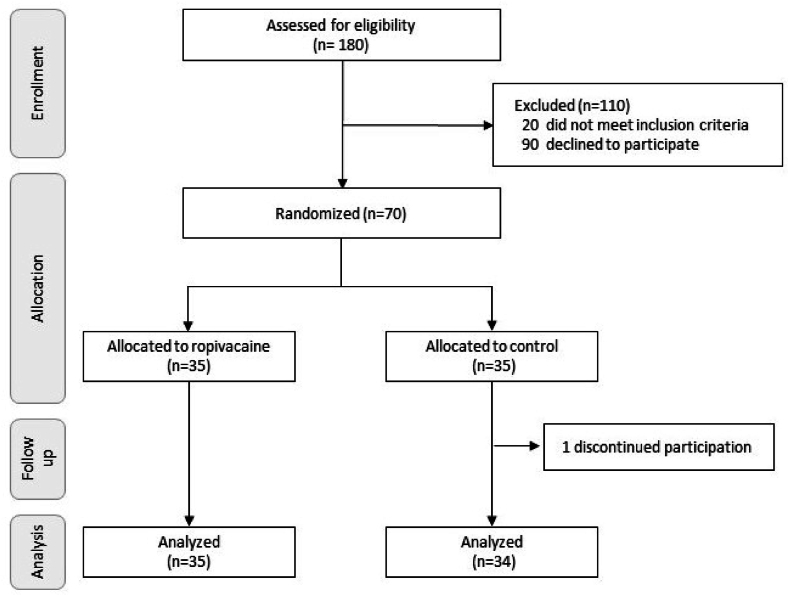
Flowchart showing the number of patients initially selected and randomized into two groups, with 35 patients in the ropivacaine group and 34 patients in the control group (one patient withdrew her participation).

A total of 70 patients were initially recruited in the study, including 35 in the study group and 35 in the control group. Fig. 1 provides a Consolidated Standards of Reporting Trials (CONSORT) diagram of the study. After cesarean section, one patient assigned to the control group withdrew from the study. Therefore, the final analysis was done in 69 patients (35 in the study group and 34 in the control group).

The general characteristics of patients showed no difference in age, multiparity, maternal BMI, preoperative vital signs and underlying disease (Table 1). The study group underwent fewer cases of general anesthesia (25 cases 71.43 % in the study group, 32 cases 94.12 % in the control group, p = 0.0129).

**Table 1:** General characteristics of patients.

	Study group (n = 35)	Control group (n = 34)	*p* value
Age (y)	33.03 ± 3.78	32.18 ± 6.30	0.9376
Multiparous (case)	17 (48.57 %)	18 (52.94 %)	0.6348
Maternal BMI (kg/m^2^)	28.69 ± 3.88	28.82 ± 4.40	0.8973
Systolic BP (mmHg)	127.69 ± 17.27	126.09 ± 15.43	0.9473
Diastolic BP (mmHg)	76.80 ± 11.22	78.41 ± 12.34	0.5723
Smoking	35 (100.00 %)	32 (94.12 %)	0.2391
Alcohol	32 (91.43 %)	34 (100.00 %)	0.2391
Repeat cesarean section			0.2421
0	23 (65.71 %)	19 (55.88 %)	
1	12 (34.29 %)	12 (35.29 %)	
2	0 (0.00 %)	3 (8.82 %)	
Pregestational diabetes	32 (91.43 %)	33 (97.06 %)	0.6139
Chronic hypertension	33 (94.29 %)	34 (100.00 %)	0.4928
Anesthesia methods General anesthesia Spinal anesthesia	25 (71.43 %) 10 (28.57 %)	32 (94.12 %) 2 (5.88 %)	0.0129

Continuous variables: Age (y), maternal BMI (kg/m^2^), systolic BP (mmHg), diastolic BP (mmHg); Wilcoxon rank-sum test or independent sample *t*-test, Categorical variables: Multiparous, smoking, alcohol, repeat cesarean section, pregestational diabetes, chronic hypertension; Fisher's exact test or Chi-square test. BMI, body mass index; BP, blood pressure.

Among the pregnancy outcomes, gestational age at delivery was earlier in the control group because there were more cases of preterm premature rupture of membranes (PPROM) in the control group (Table 2, Table 3). In the newborns, the median value of the Apgar scores at 5 min was the same for both groups (median value: 9, with a range of 4–10 in the study group and 4–9 in the control group) (Table 2).

**Table 2:** Pregnancy outcomes.

	Study group (n = 35)	Control group (n = 34)	*p* value
Gestational age at delivery			0.0179
mean ± SD	37.60 ± 1.83	36.26 ± 2.93	
min, max	30, 40	25, 40	
Neonate birth weight(g)			0.1313
mean ± SD	3022 ± 592	2776 ± 739	
min, max	1475, 4260	755, 3950	
Apgar score at 5 min			0.2814
mean ± SD	8.51 ± 1.09	8.18 ± 1.31	
median (1st IQ–3rd IQ)	9 (8–9)	9 (8–9)	
min, max	4, 10	4, 9	

SD, standard deviation; IQ interquartile.

**Table 3:** Obstetric complications duplicate outcomes.

	Study group (n = 35)	Control group (n = 34)	*p* value
Obstetric complications			0.3262
Yes^†^	5 (14.29 %)	8 (23.53 %)	
No	30 (85.61 %)	26 (76.47 %)	
Details			
PIH	3 (8.57 %)	4 (11.76 %)	0.6661
Gestational diabetes	0(0.00 %)	3 (8.82 %)	0.0742
Placenta previa	2 (5.71 %)	2 (5.88 %)	0.9766
PPROM	2 (5.71 %)	5 (14.71 %)	0.2221
Preterm labor	2 (5.71 %)	1 (2.94 %)	0.5448
Oligohydramnios	1 (2.86 %)	0 (0.00 %)	0.3280

PIH, pregnancy induced hypertension; PPROM, preterm premature rupture of membranes.

Categorical variables: Obstetric complications; Fisher's exact test or Chi-square test.

†Include duplicates: Study group: 10 cases, Control group: 15 case.

We followed up the patients within 8 h after surgery and then on the postoperative days 1 and 2. The duration of the first visit after surgery was not different between the two groups (5.77 h ± 1.66 h for the study group and 5.37 h ± 2.29 h for control; p = 0.5478).

**Fig. 2:**
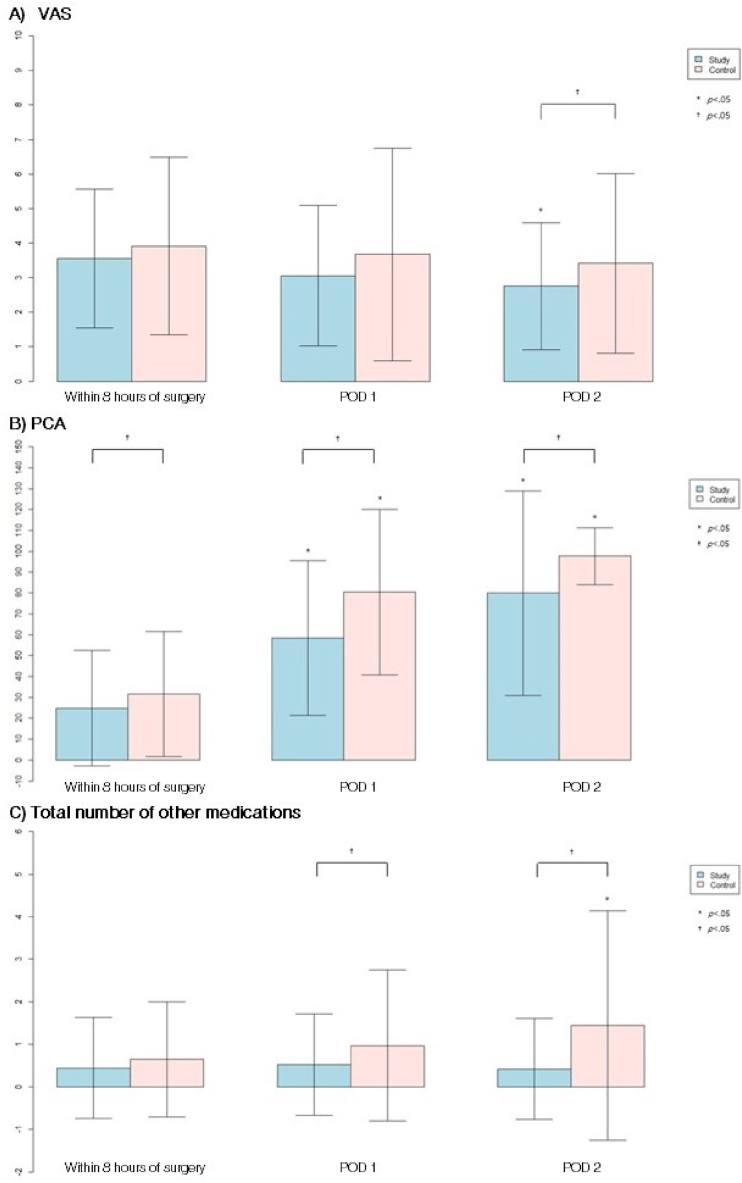
Primary and secondary outcome. Pain was evaluated less than 8 h after surgery, POD 1 and POD 2: A, corresponding to the intensity of pain on visual analog scale(VAS, 0–10) after cesarean section; B, total volume of analgesic infused by patient-controlled analgesia (PCA) pump; C, total number of non-opioid medications used postoperatively. PCA, patient-controlled analgesia; POD, postoperative day; VAS, visual analogue scale. ^†^Comparison between ropivacaine group and control group. ∗Comparison within the group, between within 8 h and either POD 1 or POD 2.

The mean value of VAS (primary outcome) was not different between the groups at the first visit, but it was lower in the study group at POD1 and became significantly lower at POD 2 compared to that in the other group (p = 0.0287, Table 4 and Fig. 2)

**Table 4:** Primary and secondary endpoints.

1. Primary endpoint: VAS (visual analogue scales)			
	Study group (n = 35)	Control group (n = 34)	*p* value^a^
<8 h	3.57 ± 1.04	3.91 ± 1.31	0.2931
POD 1 *p* value^b^	3.09 ± 1.04 0.0935	3.68 ± 1.57 0.5829	0.0907
POD 2 *p* value^c^	2.74 ± 0.95 0.0034∗	3.41 ± 1.33 0.0884	0.0287

2. Secondary endpoint 1) Total volume infused by patient-control analgesics (ml)			
Within 8 h	24.43 ± 14.08	35.15 ± 16.94	0.0047
POD 1	58.97 ± 21.07	83.09 ± 23.19	<0.001
POD 2	86.57 ± 25.23	110.88 ± 20.36	<0.001
2) Total number of additional analgesics			
Within 8 h	0.46 ± 0.61	0.65 ± 0.69	0.2433
POD 1 *p* value^b^	0.54 ± 0.11 0.5454	0.97 ± 0.90 0.0567	0.0477
POD 2 *p* value^c^	0.49 ± 0.74 1.0000	1.44 ± 1.42 0.0018	0.0029
3) Other complications (N, %)			
PONV	3 (8.82 %)	1 (2.94 %)	0.3098
Dizziness	5 (14.71 %)	4 (11.76 %)	0.7253

POD, postoperative day, PONV, postoperative nausea and vomiting.

a Differences between groups, Wilcoxon rank-sum test or independentsample *t*-test.

b Comparison within the group between within 8 h and POD 1.

c Comparison within the group between POD 1 and POD 2.

The secondary outcomes were the requirements for opioid PCA and other analgesics (Table 4 and Fig. 2). Fentanyl-based PCA usage was lower in the study group at all visits compared to the other group, and over time, the difference between the two groups became statistically significant 24 h after surgery (Table 4 and Fig. 2). Frequency of administration of additional analgesics, sequentially in the order of propacetamol, ketorolac, and pethidine hydrochloride, was higher in the control group compared to that in the study group and differed significantly between the two groups from the second visit. In six patients, pethidine hydrochloride was additionally administered seven times (six patients in the control group and one patient in the study group).

There was no significant difference in the rate of PONV and dizziness between the groups (Table 4).

## 4 Discussion

In our study, ropivacaine wound infusion reduced the requirements for fentanyl-based PCA and other analgesics and improved pain scores when compared to saline wound infusion. Wound infusion with ropivacaine was effective in controlling postoperative pain, and this effect became apparent over time as the gap between the VAS scores of the two groups widened. Regarding the use of painkillers, the study group had lower requirements for fentanyl-based PCA and other analgesics than the saline control had.

A systematic review conducted by Bamigboye and Hofmeyr found that using wound infiltration with anesthetics after cesarean section decreased morphine consumption by 1.70 mg at 24 h when compared with using placebo [5]. Adesope et al. suggested that local wound infiltration with anesthetics decreased postoperative opioid consumption [6]. In our study, continuous infusion with local anesthetics reduced total 200 μg of fentanyl citrate during IV-PCA use at the 48 h after surgery (Table 4).

Post-operative pain immediately after cesarean section is known to be severe and intense regardless of the type of surgical procedure [10]. Adequate pain control can improve the prognosis of both the mother and newborn; therefore, various analgesia techniques are used in this obstetric population. Wound perfusion with local anesthetics is the first used technique for postoperative pain after thoracotomy [7], and using the On-Q pump has been attempted for reducing pain and opioid requirement after cesarean section.

In the early studies of continuous wound infusion, where the catheter was inserted into the subcutaneous layer, the opioid requirements were reduced but the VAS score was not improved by local anesthetics in comparison to placebo [11,12]. According to the findings of Rackelboom et al., the exact location of the anatomical layer is important for the effect of the continuous wound infusion of ropivacaine combined with non-steroidal anti-Inflammatory drugs (NSAIDs), compared to infusion above the fascia, significantly reduced both pain at rest and total opioid consumption [13]. In this study, the catheter location for infusion was the subfascial layer, consistent with previous reports that the catheter should be placed subfascially rather than subcutaneously for optimal efficacy.

When comparing the nature of intrathecal opioids, one study showed that continuous ropivacaine in-wound infusion was less effective in postoperative pain control than that with intrathecal morphine [14]. However, this finding may be partially explained by the subcutaneous layer placement of the multi-holed catheter. In terms of postoperative pain control efficacy, subfascial continuous wound infusion with local anesthetics is comparable to neuraxial opioid injection [[15], [16], [17]], with significantly less side effects [17].

Jolly et al. reported that continuous levobupivacaine subfascial infiltration without subarachnoid morphine compared with placebo reduced morphine consumption through PCA (6.7 mg, p = 0.020) and intensity of postoperative pain [16]. Furthermore, Lalmand et al. [15] compared the time to first morphine request to investigate the duration of analgesia, in three groups receiving either intrathecal morphine, local anesthetics through a catheter, or saline control after cesarean section. They found that both the intrathecal morphine and local anesthetics groups experienced a significantly longer analgesic duration and lower cumulative morphine consumption compared with those in the control group.

In our study, continuous wound infusion with ropivacaine was well tolerated in patients until catheter removal on POD 2. There was no catheter-related infection and pump failure, but the inserted catheter might itself cause pain, as some patients felt relieved after its removal. Regarding the infusion medication, ropivacaine was selected for this study because ropivacaine showed lower systemic toxicity and shorter half-life compared with either bupivacaine or levobupivacaine, and no case of local anesthetics-related toxicity was observed.

Some prior studies have demonstrated enhanced pain relief with the inclusion of NSAIDs alongside local anesthetics in continuous wound infusion [8,18]. Wagner-Kovacec et al. showed that adding ketorolac to continuous wound infusion with bupivacaine significantly improved analgesia after cesarean section and decreased opioid consumption when compared to bupivacaine infusion alone [8]. The benefit of this combination was supported by a study showing the reduction of inflammatory cytokines (Interleukins 6 and 10) in the wound exudate [18].

As standard care after cesarean section, a multimodal approach to analgesia is recommended by experts and the professional associations [4,19,20]. This approach aims not only to achieve adequate pain relief, but also to reduce the prescription requirements for opioids. Recently, the death rate among reproductive-aged women due to opioid-use disorders has been increasing in the United States, with most of the cases initially exposed to prescription opioids during common medical events such as cesarean delivery [21,22]. In a study of opioid use among postpartum women, patients who did not dispense opioids in the immediate post-delivery period were less likely of chronic opioid use for the year following birth-giving [22]. Furthermore, considering the potential transfer of opioid analgesics to breastfeeding infants, prescription opioids should be reserved for the breakthrough pain management when the earlier combination of neuraxial opioids and non-opioid analgesics is inadequate. The amounts of drugs in breast milk reflects the maternal blood levels. Clinicians are advised to use the minimal efficacious dosage of opioids and prefer intrathecal or epidural administration over intravenous delivery whenever feasible.

The limitations of this study include the single-institutional design and absence of neuraxial anesthesia and intrathecal morphine use unlike the previous studies. Most patients underwent surgery under general anesthesia due to the anesthesiology department affiliation, and more patients usually demand general anesthesia out of fear during surgery. However, neuraxial anesthesia is more strongly promoted as the standard analgesia for cesarean section than general anesthesia because of the decreased maternal risk and improved fetal outcomes, with decreased pain through intrathecal opioid injection right after surgery [4,23]. In contrast to previous, this study showed that wound infusion was also effective in patients under general anesthesia. The subgroup analysis by the kind of anesthesia revealed the ropivacaine wound infusion was significantly effective in the pain relief and analgesics reduction of the patient under general anesthesia (the supplementary data).

Additionally, multiple anesthesia teams were involved, which might affect the consistency of anesthesia type and level of pain right after surgery across the patients. While the cesarean sections were performed by a single surgeon, the variability in anesthesia personnel introduced an element of heterogeneity, which should be considered when interpreting the results. Further study is needed on evaluating the effectiveness of wound infusion in patients according to the kind of anesthesia, the impact on implementation of breastfeeding, and the extent of analgesia according to the kind of drugs infused.

In conclusion, the continuous infusion of ropivacaine into the subfascial wound via a multi-hole catheter leads to reduced postoperative opioid usage and pain scores. This method may serve as an effective approach for pain management following cesarean section, without elevating the occurrence of adverse effects.

## CRediT authorship contribution statement

**Woo Jeng Kim:** Writing – original draft, Visualization. **Eui-Jin Cho:** Visualization. **Gyul Jung:** Methodology, Investigation. **In Seon Hwang:** Software, Formal analysis. **Jong Bun Kim:** Resources, Data curation. **Yoonho Kim:** Resources, Data curation. **Hee Joung Lee:** Validation, Software. **Yeon-Hee Kim:** Writing – review & editing, Writing – original draft, Supervision, Project administration, Methodology, Investigation, Funding acquisition, Conceptualization.

## Ethical statement

The Uijeongbu St. Mary's Hospital ethical review board approved the study (approval number UC20DISI0042). Written informed consent was obtained from the patients, and the study was carried out in accordance with the principles of the Declaration of Helsinki.

## Clinical trial

The clinical trial descried in this paper was registered at Clinical Research Information Service (CRiS), Republic of Korea under the registration number KCT0005108.

## Funding

Yeon-Hee Kim received research grants from B. Braun Korea. B. Braun Korea was not involved with this work in any manner. The other authors did not report any potential conflicts of interest.

## Declaration of competing interest

The authors declare the following financial interests/personal relationships which may be considered as potential competing interests:Yeon-Hee Kim reports financial support was provided by B. Braun Korea. If there are other authors, they declare that they have no known competing financial interests or personal relationships that could have appeared to influence the work reported in this paper.

## Data Availability

Research data can be checked through the following link; https://cris.nih.go.kr/cris/search/detailSearch.do?seq=27261&status=5&seq_group=16613&search_page=M Moreover, the data is available upon request (yoni@catholic.ac.kr).
