# Supplementary material for: Efficacy of continuous preperitoneal ropivacaine infusion in women with cesarean section: A prospective, randomized controlled, single blinded study
Source: Heliyon. 2024 Oct 21;10(21):e39608. doi: 10.1016/j.heliyon.2024.e39608 (PMC11546453; doi:10.1016/j.heliyon.2024.e39608)
Supplement: Multimedia component 1 [file mmc1.docx]

***Supplementary data***

**Table 1.1.** Primary and secondary endpoints(General anesthesia patients)

| 1. Primary endpoint: VAS (visual analogue scales) | | | |
| --- | --- | --- | --- |
|  | Study group (n=25) | Control group (n=32) | *p* value^a^ |
| Within 8 hours | 3.44 ± 1.04 | 3.91 ± 1.33 | 0.1436 |
| POD 1  *p* value^b^ | 3.00 ± 1.15  0.1976 | 3.75 ± 1.59  0.6040 | 0.0437* |
| POD 2  *p* value^c^ | 2.72 ± 1.02  0.0308* | 3.44 ± 1.34  0.0788 | 0.0258* |
| 1. Secondary endpoint 2. Total volume infused by patient-control analgesics (ml) | | | |
| Within 8 hours | 24.00 ± 14.43 | 31.16 ± 17.44 | 0.0107* |
| POD 1 | 59.96 ± 23.36 | 83.59 ± 23.80 | 0.0004* |
| POD 2 | 84.00 ± 27.88 | 111.56 ± 20.81 | 0.0002* |
| 1. Total number of additional analgesics | | | |
| Within 8 hours | 0.48 ± 0.59 | 0.66 ± 0.70 | 0.3059 |
| POD 1  *p* value^b^ | 0.48 ± 0.59  1.0000 | 0.94 ± 0.91  0.0951 | 0.0258* |
| POD 2  *p* value^c^ | 0.48 ± 0.77  1.0000 | 1.34 ± 1.38  0.0035* | 0.0043* |

^a^Differences between groups, Wilcoxon rank-sum test or independent sample t-test.

^b^Comparison within the group between within 8 hours and POD 1.

^c^Comparison within the group between POD 1 and POD 2.

**Table 2.2.** Primary and secondary endpoints(Spinal anesthesia patients)

| 1. Primary endpoint: VAS (visual analogue scales) | | | |
| --- | --- | --- | --- |
|  | Study group (n=10) | Control group (n=2) | *p* value^a^ |
| <8 hours | 3.90 ± 0.99 | 4.00 ± 1.41 | 0.9373 |
| POD 1  *p* value^b^ | 3.30 ± 0.67  0.1114 | 2.50 ± 0.71  0.5000 | 0.3270 |
| POD 2  *p* value^c^ | 2.80 ± 0.79  0.0175* | 3.00 ± 1.41  0.7048 | 0.8751 |
| 1. Secondary endpoint 2. Total volume infused by patient-control analgesics (ml) | | | |
| Within 8 hours | 25.50 ± 13.83 | 35.00 ± 7.07 | 0.2503 |
| POD 1 | 56.50 ± 14.54 | 75.00 ± 7.07 | 0.0684 |
| POD 2 | 93.00 ± 16.36 | 100.00 ± 0.00 | 0.2091 |
| 1. Total number of additional analgesics | | | |
| Within 8 hours | 0.40 ± 0.70 | 0.50 ± 0.71 | 0.8775 |
| POD 1  *p* value^b^ | 0.70 ± 0.67  0.3434 | 1.50 ± 0.71  - | 0.3270 |
| POD 2  *p* value^c^ | 0.50 ± 0.71  0.7263 | 3.00 ± 1.41  - | 0.2286 |

^a^Differences between groups, Wilcoxon rank-sum test or independent sample t-test.

^b^Comparison within the group between within 8 hours and POD 1.

^c^Comparison within the group between POD 1 and POD 2.
